# Cerebrospinal fluid A beta 1–40 peptides increase in Alzheimer’s disease and are highly correlated with phospho-tau in control individuals

**DOI:** 10.1186/s13195-020-00696-1

**Published:** 2020-10-02

**Authors:** Sylvain Lehmann, Julien Dumurgier, Xavier Ayrignac, Cecilia Marelli, Daniel Alcolea, Juan Fortea Ormaechea, Eric Thouvenot, Constance Delaby, Christophe Hirtz, Jérôme Vialaret, Nelly Ginestet, Elodie Bouaziz-Amar, Jean-Louis Laplanche, Pierre Labauge, Claire Paquet, Alberto Lleo, Audrey Gabelle, for the Alzheimer’s Disease Neuroimaging Initiative (ADNI)

**Affiliations:** 1 Univ Montpellier, CHU Montpellier (LBPC-PPC), INSERM (IRMB, INM), Montpellier, France; 2 Centre de Neurologie Cognitive et Service de Biochimie et de Biologie Moléculaire, Groupe Hospitalier Lariboisière Fernand-Widal, INSERMU942, Université Paris Diderot, Paris, France; 3 CHU de Montpellier, Département de Neurologie, INSERM, Univ Montpellier, Montpellier, France; 4 Sant Pau Memory Unit, Department of Neurology, Universitat Autònoma de Barcelona, Barcelona, Spain; 5 CHU de Nîmes, Département de Neurologie, INSERM, Univ Montpellier, Montpellier, France; 6 Univ Montpellier, INSERM, CHU Montpellier (CMRR), Montpellier, France

**Keywords:** Alzheimer’s disease, Amyloid peptides, Tau proteins, Biomarkers, Cerebrospinal fluid (CSF)

## Abstract

**Background:**

Amyloid pathology, which is one of the characteristics of Alzheimer’s disease (AD), results from altered metabolism of the beta-amyloid (Aβ) peptide in terms of synthesis, clearance, or aggregation. A decrease in cerebrospinal fluid (CSF) level Aβ1–42 is evident in AD, and the CSF ratio Aβ42/Aβ40 has recently been identified as one of the most reliable diagnostic biomarkers of amyloid pathology. Variations in inter-individual levels of Aβ1–40 in the CSF have been observed in the past, but their origins remain unclear. In addition, the variation of Aβ40 in the context of AD studied in several studies has yielded conflicting results.

**Methods:**

Here, we analyzed the levels of Aβ1–40 using multicenter data obtained on 2466 samples from six different cohorts in which CSF was collected under standardized protocols, centrifugation, and storage conditions. Tau and p-tau (181) concentrations were measured using commercially available in vitro diagnostic immunoassays. Concentrations of CSF Aβ1–42 and Aβ1–40 were measured by ELISA, xMAP technology, chemiluminescence immunoassay (CLIA), and mass spectrometry. Statistical analyses were calculated for parametric and non-parametric comparisons, linear regression, correlation, and odds ratios. The statistical tests were adjusted for the effects of covariates (age, in particular).

**Results:**

Regardless of the analysis method used and the cohorts, a slight but significant age-independent increase in the levels of Aβ40 in CSF was observed in AD. We also found a strong positive correlation between the levels of Aβ1–40 and p-tau (181) in CSF, particularly in control patients.

**Conclusions:**

These results indicate that an increase in the baseline level of amyloid peptides, which are associated with an increase in p-tau (181), may be a biological characteristic and possibly a risk factor for AD. Further studies will be needed to establish a causal link between increased baseline levels of Aβ40 and the development of the disease.

## Background

Alzheimer’s disease (AD) neuropathological brain lesions consist of aggregates of hyper-phosphorylated tau proteins, which have also been called neurofibrillary tangles (NFTs), and extracellular deposits of amyloid precursor protein (APP) derived amyloid-beta (Aβ) peptides, which are known as amyloid plaques. Much research has been focusing recently on the molecular mechanisms underlying these pathological events as it has become essential to develop preventive and therapeutic strategies for AD. For a long time, the main explanation for the pathogenesis of AD was that amyloidogenesis was the *primum movens* of the affection, which led to the concept of the amyloid cascade [1]. According to this picture of the disease, the alteration of APP metabolism (increasing amyloid production, decreasing clearance rates), the aggregation of Aβ peptides, and the formation of amyloid plaques might result in microglial and astrocyte activation, local inflammatory responses, oxidative stress, and eventually the hyper-phosphorylation of tau proteins and secondarily the formation of NFTs [2].

The idea that amyloid peptides contribute importantly to the etiology of AD is supported by cases of AD who carry presenilins (1 or 2) or APP mutations [3]. These gene mutations trigger the overproduction of Aβ peptides or the preferential production of Aβ42, which is the most amyloidogenic of all the peptides. An APP gene dose effect triggering AD development, as occurs in Down syndrome [4] and in gene duplication processes [5], is a further/an additional potential factor contributing to amyloid pathogenesis. Other genetic factors have been described, such as apolipoprotein E4 allele, in particular [6].

Studies on cerebrospinal fluid (CSF) biomarkers in AD have greatly improved our understanding of the pathophysiology of this disease. The production of amyloid peptides following the neuronal processing of APP has been involved in the response to physiological challenge with neurotrophic, anti-microbial, tumor suppression, or synaptic function regulation activities [7]. Regarding tau proteins which are associated to microtubules, their physiological secretion by neuronal cells is a recent discovery which physiological relevance and benefit are still matter of debate [8]. A decrease in CSF Aβ42 is especially indicative of an amyloidogenic process, while an increase in tau proteins (total tau and its phosphorylated form p-tau (181)) is known to be associated with axonal loss and tau pathology in AD [9, 10]. Tests on these two biomarkers are being included nowadays in the international clinical research guidelines [11, 12], and many centers [11–15], and ourselves [13–15] have integrated them into daily clinical practice. Importantly, these biochemical CSF measurements are concordant with the results of the PET imaging approaches which were initially developed to determine the brain amyloid load [16], and now also serve to measure tau accumulation [17]. These data are in line with hypotheses put forward by Jack et al. [18] about the chronology of the evolution of biomarkers during the pathophysiological process, and the relevance of amyloid markers in particular at a very early stage, probably as early as 10 to 15 years before the onset of clinical symptoms.

Under non-pathological conditions, Aβ40 is highly correlated with Aβ42 [19]. The computation of the ratio Aβ42/40 is now being used in routine clinical practice on AD patients in some centers [20–22]. This is a useful approach for reducing pre-analytical Aβ42 biases [23–25] and improving the diagnostic performances of CSF biomarkers [26], especially in discordant cases [27]. This ratio can also be used to account for inter-individual amyloid variations in the baseline CSF level [28]. Low CSF Aβ40 levels might also be indicative of frontotemporal dementia (FTD) [29, 30], cerebral amyloid angiopathy (CAA) [31], HIV [32], multiple sclerosis [33], or normal pressure hydrocephalus (NPH) [34].

In their meta-analysis of AD biomarkers, Olsson et al. [35] observed the existence of a negligible difference in CSF Aβ40 between AD and control patients. Most of the 32 studies considered had a limited number of patients included in each group (the median number of subjects per group was less than 30, and the maximum number of subjects was 137 and 328 in the AD and non-AD groups, respectively). The focus of these studies was also quite different, looking at the diagnostic interest of Aβ42/40 in AD and of Aβ peptides in other neurodegenerative diseases, or being more interested in pathophysiological mechanisms.

The rationale for this study is therefore based on several elements: first, to resolve the controversy conflicting studies showing or not an increase in Aβ40 in AD; second, to provide explanations for the very good diagnostic performance of the Aβ42/40 ratio calculation; and finally, in the context of the pathophysiology of the disease, to provide elements linking, in sporadic AD, the level of expression of amyloid peptides and the disease. We revisited the issue of the Aβ40 levels using large series of multicenter data. The results show the occurrence of a significant age-independent increase in CSF Aβ40 in AD. Another noteworthy finding was the existence of a strong positive correlation between CSF Aβ40 and the p-tau (181) concentration, even in patients without Alzheimer’s disease (NAD). These findings suggest that the baseline amyloid peptide level may constitute a risk factor contributing to sporadic AD, which is associated with p-tau (181) production.

## Methods

### Study design and subjects

Patients with cognitive impairments were recruited and followed at the Montpellier and Paris Memory Resources Center (CMRR). The Montpellier participants were subdivided into two cohorts which were recruited during different periods: Montpellier 1 (Mtp-1) (recruited from July 2015 to May 2017) and Montpellier 2 (Mtp-2) (recruited from September 2009 to June 2015). These two periods corresponded to the use of different ELISA kits (SupTable 1). The cohort Mtp-1 consisted of 400 patients (126 AD, 274 NAD), and the cohort Mtp-2 consisted of 504 patients (220 AD, 284 NAD). The Paris cohort consisted of 624 patients (299 AD, 325 NAD) from the Centre de Neurologie Cognitive, Groupe Hospitalier Lariboisière Fernand-Widal (recruited from March 2012 to May 2017). The Barcelona SPIN (Sant Pau Initiative on Neurodegeneration) cohort (79 AD, 148 NAD) consisted of patients who had undergone lumbar puncture for CSF AD biomarkers at the Sant Pau Memory Unit [36, 37] (recruited from May 2009 to December 2017). All the patients underwent a thorough clinical examination including biological lab tests, neuropsychological assessments, and brain imaging. The same diagnostic procedure [27] and AD diagnostic criteria [11] were used at all the clinical centers which participated. We included in the AD group patients which, in the absence of substantial concomitant cerebrovascular disease, meet the AD core clinical criteria and are considered as probable AD or possible AD with biomarker evidence of AD (CSF amyloid, tau, FDG-PET, or structural MRI) [11]. Although CSF amyloid biomarkers were included in the diagnostic criteria for AD, we observed that between 2 and 7% of AD cases, depending on the cohort, were not based on evidence of amyloid biomarkers but rather on other elements such as MRI. The possible AD category without amyloid or tau biomarker evidence of AD corresponds to the mild cognitive impairment (MCI) in the absence of core features of synucleinopathies, frontotemporal dementia (FTD), primary progressive aphasia, or evidence for another concurrent, active neurological disease, or a non-neurological medical comorbidity or use of medication that could have a substantial effect on cognition. Subjective cognitive impairment (SCI) corresponds to a situation where a patient reports an alteration of their cognitive functions, including memory, but this cannot be documented by clinical, neuropsychological, imaging, or biological tests.

The NAD diagnosis included SCI, CAA, NPH, FTD based on relevant criteria [38], dementia with Lewy bodies (based on the McKeith criteria [39]), corticobasal degeneration (based on the criteria defined by Boeve et al. [40]), progressive supranuclear palsy, amyotrophic lateral sclerosis, and vascular dementia (based on the usual consensus diagnostic criteria). All the patients at each clinical center gave their written informed consent to participating in clinical research on CSF biomarkers, which was approved by the respective Ethics Committees. The committee responsible in Montpellier was the regional Ethics Committee of the Montpellier University Hospital and Montpellier CSF-Neurobank #DC-2008-417 at the certified NFS 96-900 CHU resource center BB-0033-00031, www.biobanques.eu. Authorization to handle personal data was granted by the French Data Protection Authority (CNIL) under the number 1709743 v0.

Two sets of data originating from the analysis of CSF samples at the Alzheimer’s Disease Neuroimaging Initiative (ADNI) database (www.loni.ucla.edu/ADNI) were used after the agreement of the scientific committee. ADNI UPEN-RESULTS, UPEN-ELYCYS (*n* = 311), and MS UPENNMSMSABETA (*n* = 400) data were also used. In the ADNI cohorts, which included many patients with mild cognitive impairments (MCI), we had to rely on the biological PLM (Paris-Lille-Montpellier) scale [41] to define populations with a low (ADNI(−)) and high (ADNI(+)) prevalence of AD. This scale combines the concentration of the three CSF biomarkers [Aβ42, tau, p-tau (181)] into a probability scale for AD. The score ranges from 0 to 3 based on the number of abnormal CSF biomarkers. ADNI(−) population corresponded to PLM scale of 0 or 1 with less than 25% of AD, while ADNI(+) corresponded to PLM scale of 2 and 3 with more than 75% of AD. Importantly, the PLM score used was not based on the Aβ40 values so as to prevent circular reasoning. This way of stratifying patients in the ADNI cohort represents anyway a limitation of our study.

### CSF samples and assays

CSF was collected using standard conditions of collection, centrifugation, and storage [42, 43]. CSF tau and p-tau (181) concentrations were measured using the standardized commercially available INNOTEST_R_ sandwich ELISA, Luminex® xMAP technology (x = analyte, MAP = Multi-Analyte Profiling) assays in line with the manufacturer’s instructions (Fujirebio-Europe). The consistency of the p-tau (181) detection using the ELISA assays is ensured by its comparison with the mass spectrometry detection performed in this fluid [44]. In the Mtp-1 cohort, CSF Aβ1–42 and Aβ1–40 (denoted here by Aβ42 and Aβ40) were measured with Euroimmun kits (EQ-6511-9601 (Aβ1–40); EQ-6521-9601 (Aβ1–42)). In the Mtp-2 and Paris cohorts, CSF Aβ42 and Aβ40 were measured using INNOTEST_R_ sandwich ELISA from IBL and Fujirebio, respectively, as recommended by the manufacturer. Roche Elecsys automated chemiluminescence immunoassay (CLIA) and mass spectrometry were used on the ADNI cohorts to measure CSF Aβ1–42 and Aβ1–40 as previously described [45, 46]. Detection limits of these kits are compatible with CSF clinical ranges. Average concentration of analytes may differ between kits in relation with standard value assignments by the vendors in the absence of certified reference materials.

The pre-analytical procedure was standardized [43] but differed, depending on the type of collection tubes used [36, 47]. This explains the differences observed between cohorts in the mean Aβ40 and Aβ42 values measured with the same detection kit (SupTable 1). The quality of the results was ensured by using validated standard operating procedures and internal quality controls (QCs). The QC coefficient of variation obtained on the CSF analytes in each batch and between batches ranged consistently below 15%. In addition, external QC procedures were used to confirm the quality/accuracy of the results [42]. In the case of the ADNI cohorts, CSF samples were deep frozen after the lumbar puncture without performing any centrifugation or aliquoting, and shipped to the UPENN ADNI Biomarker Laboratory in Philadelphia on dry ice, where they were thawed, aliquoted, and re-frozen.

### Statistical analysis

Statistical analyses were computed with the MedCalc software program (18.11.3). Data tested for normality were expressed either as means ± SDs or as median 25th/75th percentile, and differences between groups were taken to be significant in the Student *t* tests or the non-parametric Mann-Whitney *U* test at *P* < 0.05. Linear regression was computed between continuous biomarkers, and the corresponding Pearson correlation coefficients and statistical significance have been specified in the tables. For meta-analysis of the study, we used the Hedges *g* statistic as a formulation for the standardized mean difference (SMD) under the fixed effects model. The SMD Hedges *g* is the difference between the two means divided by the pooled standard deviation. When indicated, statistical tests were adjusted to account for the effects of covariates (age, in particular). Odds ratios corresponded to the presence of AD in the various percentile groups, based on the distribution of Aβ40. The 95% confidence odds ratio intervals were computed along with the *z*-statistics and the associated *P* values.

## Results

### CSF Aβ42 and Aβ40 in AD and NAD populations

**Fig. 1:**
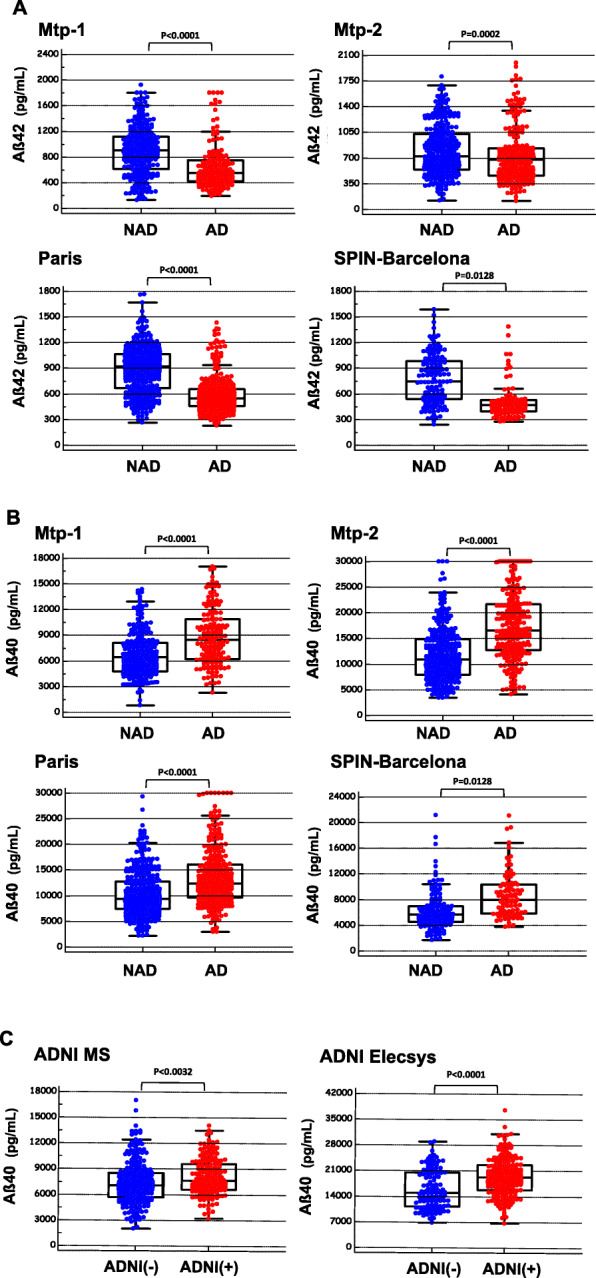
CSF Aβ42 and Aβ40 in non-AD and AD populations*.* CSF concentration of Aβ42 (**a**) and Aβ40 (**b**) in four independent cohorts (Montpellier 1 (Mtp-1), Montpellier 2 (Mtp-2), Paris, SPIN-Barcelona) confirmed the significant difference between NAD and AD patients for both analytes (*t* test). Differences in CSF Aβ40 measured in two ADNI cohorts (**c**) were also significant between ADNI(+) and ADNI(−) patients stratified using the PLM scale (see the “Methods” section). Note that Aβ has been assessed using five different detection methods (supTable 1)

**Fig. 2:**
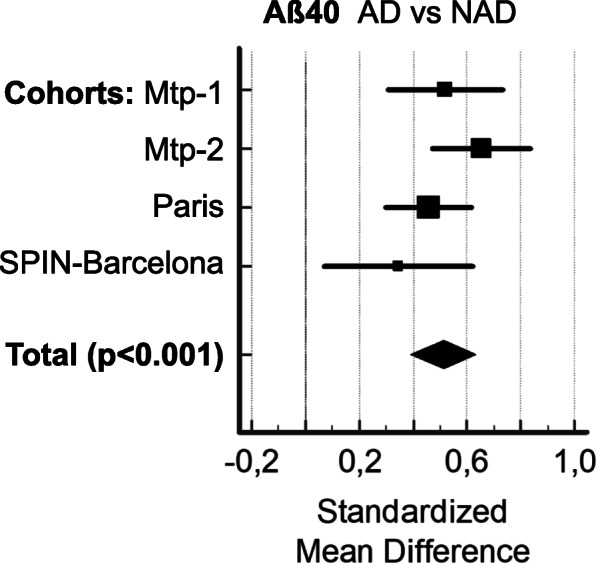
Meta-analysis including the four independent cohorts. We used with to compare the Aβ40 means between AD and NAD populations the Hedges *g* statistic as a formulation for the standardized mean difference (SMD) under the fixed effects model. The SMD Hedges *g* is the difference between the two means divided by the pooled standard deviation. The plot has marker sizes relative to study weight. The results of the different studies, with 95% CI, and the overall standardized mean difference with 95% CI are shown. Cohen’s rule of thumb for interpretation of the SMD statistic is that a value of 0.2 indicates a small effect, a value of 0.5 indicates a medium effect, and a value of 0.8 or larger indicates a large effect

CSF data on 2466 samples originating from six different cohorts were included in the present study. The AD and NAD populations were defined based on clinical criteria in the Montpellier 1 (Mtp-1), Montpellier 2 (Mtp-2), Paris, and Barcelona cohorts. For the ADNI cohort, we relied on the PLM score to distinguish ADNI(+) and ADNI(−) population. Differences were observed in terms of age and CSF biomarker profiles in the overall population, as well as in each clinical cohort (Table 1). As was to be expected, AD patients obtained lower MMSE scores and showed higher CSF tau and p-tau (181) levels than NAD patients/participants. A significant decrease in the CSF Aβ42 concentrations was observed in the AD population (Fig. 1a), regardless of the cohort tested. Noteworthy differences were also observed in the Aβ40 levels between all the cohorts (Fig. 1b): the values recorded in the AD population were significantly higher than in the NAD group regardless of analytical method, the sex, or the age as covariate. We then conducted a meta-analysis study including the four independent cohorts using the Hedges *g* statistic as a formulation for the standardized mean difference (SMD) under the fixed effects model (Fig. 2). The overall SMD with 95% CI is at 0.5, which is greater than 0.2, the significance level. The difference between AD and NAD population for Aβ40 was however limited with an important overlap resulting in AUCs lower than 0.8 (Sup Figure 2). The stratification of the ADNI population using the PLM scale (combining Aβ42, tau, and p-tau (181)) limits the interpretation of the Aβ40 results of this cohort in our study. We have nevertheless observed that the ADNI(+) group, which by definition has less Aβ42, did indeed have more Aβ40 (Fig. 1c). The physiological correlation between CSF Aβ40 and Aβ42 [19] is therefore not well preserved in AD.

**Table 1:** Demographical and cerebrospinal fluid (CSF) biomarkers’ characteristics of the six cohorts: Montpellier 1 (Mtp-1), Montpellier 2 (Mtp-2), Paris, SPIN-Barcelona, ADNI-MS, and ADNI-Elecsys

**Cohort/analyte**	**NAD**		**AD**		***P***	**Cohort/analyte**	**NAD**		**AD**		***P***
**Mtp-1**	**Mean**	**SD**	**Mean**	**SD**		**SPIN-Barcelona**	**Mean**	**SD**	**Mean**	**SD**	
Aß40	7035	2800	8579	3274	< 0.0001	Aß40	6799	3633	7985	2891	0.0128
Aß42	909	399	561	237	< 0.0001	Aß42	793	288	440	81	< 0.0001
Tau	343	279	739	267	< 0.0001	Tau	284	223	727	392	< 0.0001
p-Tau	42	16	96	36	< 0.0001	p-Tau	47	22	103	47	< 0.0001
Age	67.0	11.4	70.7	9.2	0.0015	Age	66.1	10.1	70.8	8.3	0.0004
MMSE	22.1	6.2	19.8	7.5	0.1392	MMSE	26.6	6.2	23.5	4.6	0.0097
Sex (%M)	53.6%	–	38.1%	–	0.0040*	Sex (%M)	57.4%	–	38.0%	–	0.0055*
ApoE (% E4)	NA					ApoE (% E4)	63.3%	–	49.4%	–	0.1910*
**Cohort/analyte**	**NAD**		**AD**		***P***	**Cohort/analyte**	**ADNI(−)**		**ADNI(+)**		***P***
**Mtp-2**	**Mean**	**SD**	**Mean**	**SD**		**ADNI-MS**	**Mean**	**SD**	**Mean**	**SD**	
Aß40	12,720	6046	16,802	6473	< 0.0001	Aß40	7387	2517	8101	2167	0.0032
Aß42	821	369	700	334	0.0002	Aß42	1076	547	620	231	< 0.0001
Tau	321	248	655	302	< 0.0001	Tau	66	24	140	58	< 0.0001
p-Tau	42	18	89	37	< 0.0001	p-Tau	23	8	50	18	< 0.0001
Age	66.0	12.7	70.0	9.3	0.0001	Age	75.4	6.7	74.0	7.5	0.0430
MMSE	21.9	7.3	19.6	5.4	0.0031	MMSE	27.3	2.4	25.9	2.6	< 0.0001
Sex (%M)	51.4%	–	50.9%	–	0.9114*	Sex (%M)	61.0%	–	58.6%	–	0.6349*
ApoE (% E4)	ND					ApoE (% E4)	53.8%	–	89.3%	–	< 0.001*
**Cohort/analyte**	**NAD**		**AD**		***P***	**Cohort/analyte**	**ADNI(−)**		**ADNI(+)**		***P***
**Paris**	**Mean**	**SD**	**Mean**	**SD**		**ADNI-Elecsys**	**Mean**	**SD**	**Mean**	**SD**	
Aß40	10,767	4468	13,149	5850	< 0.0001	Aß40	15,865	5265	19,417	5224	< 0.0001
Aß42	925	277	574	200	< 0.0001	Aß42	1039	610	673	269	< 0.0001
Tau	236	134	609	280	< 0.0001	Tau	241	68	433	148	< 0.0001
p-Tau	42	16	90	37	< 0.0001	p-Tau	21	6	45	18	< 0.0001
Age	65.2	9.4	70.7	8.1	< 0.0001	Age	72.9	7.3	72.8	6.3	0.9155
MMSE	23.0	5.2	21.8	5.8	0.0069	MMSE	27.1	2.4	25.4	3.6	< 0.0001
Sex (%M)	51.9%	–	38.5%	–	0.0008*	Sex (%M)	59.8%	–	49.7%	–	0.0816*
ApoE (% E4)	NA					ApoE (% E4)	49.7%	–	38.6%	–	0.1538*

Results are expressed as the mean ± standard deviation (SD). *Abbreviation*: *MMSE* Mini-Mental State Examination, *AD* Alzheimer’s disease, *NAD* non-Alzheimer’s disease, *ADNI(−)* cognitive patients with non-Alzheimer’s disease PLM profile, *ADNI(+)* cognitive patients with Alzheimer’s disease PLM profile, *P* significance level of the Student *t* test and *chi-squared test for the comparison of two proportions. Values of Aβ40, Aβ42, tau, and p-tau (181) are in picograms per milliliter

**Fig. 3:**
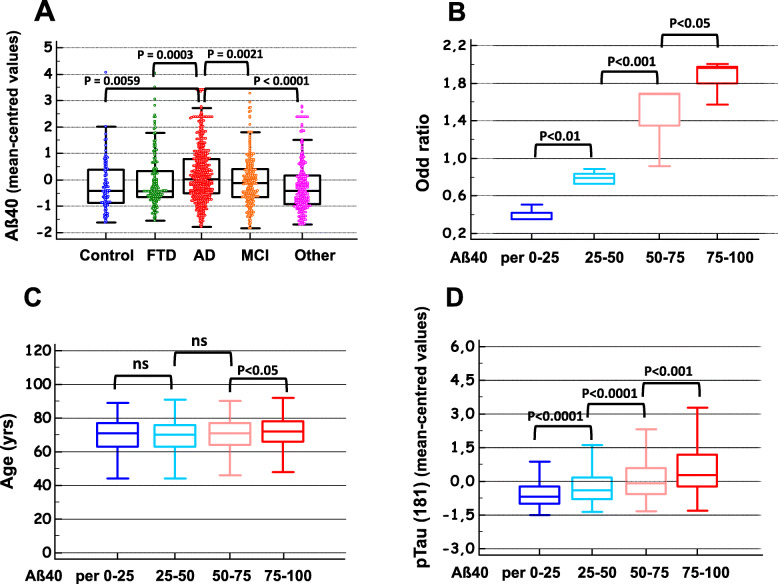
Aβ40 in different diagnoses; representation in percentile; AD odds ratio, age, and p-tau (181) distribution. The Montpellier, Paris, and SPIN-Barcelona cohorts displayed a large range of pathological samples from patients with Alzheimer’s disease (AD), mild cognitive impairment (MCI), frontotemporal dementia (FTD), Control (subjective cognitive impairment), and the other neurological diseases (Other) (see also Sup-Figure 3). Mean-centered Aβ40 values in these cohorts were combined and compared in the different clinical groups confirming the significant increase of the peptides in AD (**a**). The four cohorts (Montpellier 1 (Mtp-1), Montpellier 2 (Mtp-2), Paris, SPIN-Barcelona) have been sorted in four classes based on their Aβ40 percentile values as follows: p25, < 25th percentile; p25–50, 25th–50th percentile; p50–75, 50th–75th percentile; and p75, > 75th. The odds ratio for AD (**b**), the age of the patients (**c**), and the concentration of CSF p-tau (181) (**d**) were then plotted in each percentile class. Significant differences between classes are indicated

When we stratified and combined the clinical cohort populations into AD, MCI, FTD, Control (SCI), and other neurological diseases (Other) groups, the difference between AD and the other clinical groups was eventually confirmed (Fig. 3a). We also performed a more in-depth comparison, stratified by clinical groups. Importantly, the Paris and SPIN-Barcelona cohorts are composed of only a small number of pathologies whereas the Montpellier cohorts have a wide range of diagnoses corresponding to the Memory consultations (see the “Methods” section). As illustrated in the Sup Figure 3, we observed that AD patients have significantly higher levels of Aß40 than most other diagnostic groups, with the exception of MCI (in two out of three cohorts, there was no statistical difference between AD and MCI patients). However, this result is not unexpected since the MCI group is itself heterogeneous, with some patients progressing to AD in subsequent years. As expected from previous studies [29, 30, 48], lowered Aß40 values for CAA and FTD were found. This is even more pronounced for NPH, as expected [34]. Based on this stratification, we can conclude that the increase in Aβ40 in the AD population is not a bias related to the presence of certain diagnoses possibly present in the NAD group.

The influence of *APOE* status, which was available in the case of 983 samples (524 NAD with 36.6% E4+; 459 AD with 58.4% E4+), was also assessed with respect to the Aβ levels (SupFigure 1A-F). As previously reported [49, 50], the presence of ApoE4 was significantly associated with lower Aβ42 levels, as well as lower Aβ40 levels. The difference in Aβ40 values between NAD and AD patients was also observed in both ApoE4 positive and negative populations (SupFigure 1G-I).

To investigate more closely the relationship between Aβ40 and AD diagnosis, the total population was sorted into four percentile classes based on the value of this biomarker in the CSF (< 25th, 25th–50th, 50th–75th, and > 75th percentiles). The percentage of AD patients in each cohort clearly increased along with the Aβ40 percentiles (SupTable2). To account for the differences in AD prevalence between the cohorts, the odds ratios for AD were plotted in the case of increasing Aβ40 percentile classes, and a significant increase ranging from 0.4 to 1.8 was observed (Fig. 3b). To establish whether the difference in age observed between NAD and AD patients (SupTable 1) might be a significant determinant here, the age distribution between percentile classes was also plotted (Fig. 3c). A significant difference in age distribution was observed only between the 50th/75th and the > 75th Aβ40 percentile classes (Fig. 3c). Age cannot therefore account for the association between Aβ40 levels and AD prevalence. Nevertheless, further statistical tests were adjusted using age as covariate.

### Correlations between Aβ40 and the other CSF biomarkers

**Fig. 4:**
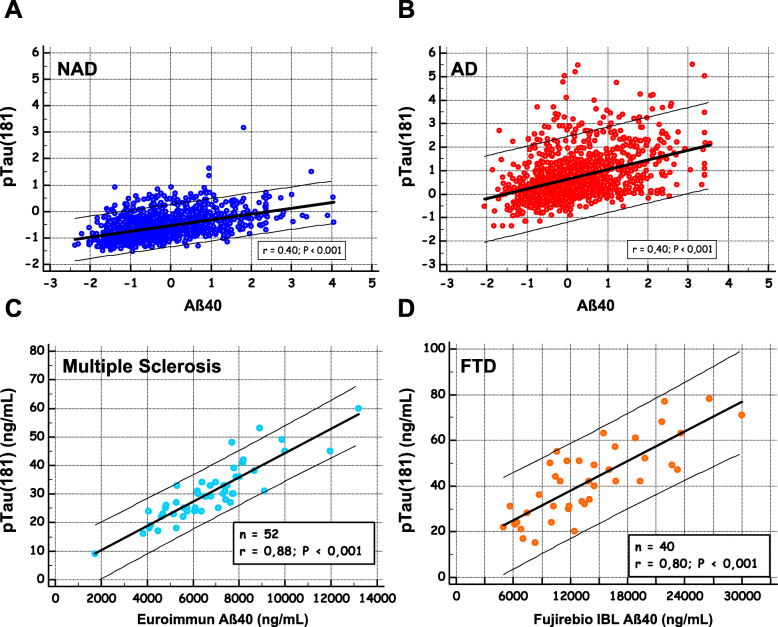
Correlation between Aβ40 and p-tau (181) in different clinical populations. To illustrate the correlation between Aβ40 and p-tau (181) (Table 2), the mean-centered concentrations of the two analytes in the total study population were plotted in NAD (**a**) and AD populations (**b**). Aβ40 and p-tau (181) concentrations were also plotted in a selection of multiple sclerosis (**c**) and FTD patients (**d**)

The correlation between Aβ40 and the other CSF biomarkers was computed in global, NAD, and AD populations, for each cohort, and in the overall population (Table 2). As was to be expected [19], a correlation was found to exist between Aβ40 and Aβ42, especially in the NAD group. Aβ40 was also correlated with the tau levels, and it was striking that the highest correlation coefficients were obtained with p-tau (181) rather than with t-tau, especially in the Mtp-1 cohort (a significant difference was observed between the correlation coefficients at *P* = 0.02). The correlation was clearly visible when the mean-centered p-tau (181) values were plotted in the various Aβ40 percentile classes (Fig. 3d), showing significant differences between classes. This correlation had to be put in perspective with the fact that both analytes increased in AD with the patients’ age (SupTable 1), which justifies the adjustments made for age in our statistical analysis. We illustrated graphically the correlation between p-tau (181) and Aβ40 in the all NAD and the AD populations (Fig. 4a, b). We noticed that p-tau (181) values were higher and more widely distributed in the AD population with a regression slope that was higher than in the NAD population. To document further the relationship between Aβ40 and p-tau (181) outside the context of AD, the correlation was tested in a series of clinically defined patients with multiple sclerosis [51] and FTD [52] (Fig. 4c, d). The corresponding correlation coefficients were both significantly higher in these groups than in the AD population (*P* < 0.001).

**Table 2:** Age-adjusted Pearson’s correlation between Aβ40 and Aβ42, tau, or p-tau (181) values in the six cohorts (Montpellier 1 (Mtp-1), Montpellier 2 (Mtp-2), Paris, SPIN-Barcelona, ADNI-MS, ADNI-Elecsys), in the overall population using mean-centered values to account for level differences between analytical methods

Aß40 correlation	All			NAD or ADNI(−)			AD or ADNI(+)		
	p-Tau	Tau	Aß42	p-Tau	Tau	Aß42	p-Tau	Tau	Aß42
**Mtp-1**	0.501	0.377	0.520	0.557	0.235*	0.764	0.504	0.443	0.613
**Mtp-2**	0.514	0.443	0.407	0.430	0.244	0.533	0.304	0.213*	0.469
**Paris**	0.419	0.36	0.188	0.421	0.145*	0.571	0.288	0.245	0.247
**SPIN-Barcelona**	0.468	0.396	0.161	0.483	0.348	0.419	0.355	0.256*	0.308*
**ADNI-MS**	0.254	0.413	0.505	0.257	0.496	0.705	0.223*	0.470	0.574
**ADNI-Elecsys**	0.536	0.567	0.449	0.487	0.624	0.782	0.502	0.496	0.580
**Overall**	0.445	0.418	0.368	0.455	0.318	0.615	0.339	0.318	0.426

Computation has been done in the All population and in the NAD, AD, ADNI(−), and ADNI(+) groups. Correlation coefficient statistical value *P* < 0.001 for all but *P < 0.01

## Discussion

The decrease in Aβ42 observed in the CSF of AD patients has attracted considerable attention in clinical and research communities. This decrease is attributable to the accumulation of Aβ42 in the brain parenchyma, along with a decrease in the rates of CSF clearance and an increase in the production of oligomeric/multimeric forms. Since determining the CSF Aβ42/Aβ40 ratio provides a useful means of improving AD diagnosis [26], many groups are now also measuring Aβ40 in their patients. A meta-analysis was however not conclusive regarding its differential levels in AD [35]. Looking back in detail at various studies, Aβ40 either was lower or showed no significant changes [21, 29, 30, 53], or apparently increased in AD in comparison with other forms of dementia [54, 55]. In a recent report, the increase in Aβ40 was clearly identified as one of the reasons for the good performances of the Aβ42/Aβ40 ratio as an index [20], while another study on PET amyloid findings also established that CSF Aβ40 increased in the PIB+ population [56]. These discrepancies might be linked to differences in cohort composition, since the Aβ40 levels may be affected by various pathological conditions [29–31]. The stage of AD, corresponding to various levels of cerebral atrophy probably reducing amyloid production [18], may also account for differences between studies. This is coherent with a recent study confirming the increase of Aβ40 in prodromal AD [57]. Differences in the precision of the analytical methods used, combined with the size of the cohorts, might also explain why only a small, non-significant difference between AD and NAD patients has been observed in some cases.

Nevertheless, the present study on CSF Aβ40 which confirms it increased in AD can be considered a “surprise.” The fact that this result has not been clearly identified previously is due to several factors, the first being that since the differences between populations are small, statistical significance requires a larger number of samples and measurement methods that are as accurate as possible. Using large cohorts, we were able to confirm the existence of an age- and ApoE-independent increase in CSF Aβ40 in AD compared with other diagnostic consisting mostly of controls and patients with other neurodegenerative diseases and dementia. This observation is valid in different analytical contexts despite differences in the threshold or range for biomarker measurement. Our finding provides additional explanations for the very good diagnostic performance of the Aβ42/40 ratio calculation [20]. Interestingly, Janelidze et al. [58] observed that some Aβ42 assays were partly quenched by Aβ40. An increase of Aβ40 in AD as suggested by our study may therefore also indirectly contribute to the diagnostic performance of Aβ42. It is worth mentioning that in the blood, where the amyloid peptide 42/40 ratio could well indicate the presence of brain amyloidosis [59, 60], it has been established that high Aβ40 levels are associated with greater mortality rate in the elderly [61]. The fact that the CSF and blood amyloid levels are poorly correlated, however, makes it difficult at this stage to extend the present conclusions to this fluid.

The overlap in the CSF Aβ40 values between the AD and NAD populations is worth noting, and the area under the receiver operating characteristic curve (AUC) for AD diagnosis was under 0.8 in all the cohorts tested (SupFigure2). CSF Aβ40 cannot therefore be used as a diagnostic biomarker but could be taken to be a feature “risk factor” in view of the odds ratio of almost 2 recorded on the population having the highest CSF Aβ40 concentration. The increased Aβ40 might be a consequence of a reduced clearance of amyloid peptides in sporadic cases and/or a higher production-lower degradation. This matches the fact that in autosomal dominant forms of AD linked to APP or presenilin mutations [3, 5] and in Down syndrome [4], an overproduction of amyloid peptides is thought to trigger the AD process, along with all its consequences, including tau protein hyper-phosphorylation, in particular.

In this context, baseline Aβ40 concentration could indicate subjects with risk of early AD development. The positive correlation found to exist in the present study between Aβ40 and p-tau (181) in AD is an additional argument supporting this pathophysiological model. Tau has many phosphorylated isoforms [62, 63], some of them believed to be more specific for AD than p-tau (181), highlighting the pathophysiological role and therapeutic interest of kinases like PKA, CAMkII, or Cdk5. This isoform is however one of the best indicators of AD pathology in the CSF where it begins to increase as two decades before the development of aggregated tau pathology [44]. In this work, we had to rely only on the correlation with p-tau (181) because it is the only isoform with in vitro diagnostic (IVD) certification and has been measured in large clinical cohorts. The fact that this correlation was also present in a control population including a subgroup of well-defined FTD [52] and multiple sclerosis patients [51] raises many questions, however. It is tempting to take this relationship to confirm that Aβ peptides may induce the phosphorylation of tau, as observed both in vitro and in vivo [64].

### Limitation

Since the present study was based on a cross-sectional design, and without neuropathological confirmation, further studies involving a longitudinal design are now required to confirm the idea that high baseline CSF levels of Aβ peptides may have prejudicial effects, leading to AD.

## Conclusions

In conclusion, our results indicate that an increase in the baseline level of amyloid peptides, which are associated with an increase in p-tau (181), could be a biological characteristic of AD. Further studies will be needed to establish a causal link between increased baseline levels of Aβ40 and the development of the disease.

## Supplementary information

**Additional file 1 : SupTable 1.** Number of patients/samples in each clinical group and Aβ40 detection method used.

**Additional file 2 : Sup-Table 2.** Percentage of AD/ADNI(+) patients in the four classes based on the Aβ40 percentile values recorded in the various cohorts; p25: 25th percentile, p25-50: 25th-50th percentile; p50-75: 50th-75th percentile; p75: 75th.

**Additional file 3 : Sup-Figure 1.** CSF *Aβ42 and Aβ40 in Non-AD and AD populations with regards to ApoE status* Mean-centered values of CSF Aβ42 (panels A-C) and Aβ40 (panels D-F) in total (all), NAD and AD population with regards to ApoE4 allele presence (+) or absence (-). Panels G-I illustrate the difference in Aβ40 between AD and NAD in the population with the ApoE status determine, in the total (all), ApoE4 allele presence (+) or absence (-).

**Additional file 4 : Sup-Figure 2.** ROC curves of *Aβ40, Aβ42 and Aβ42/40* ROC curves of Aβ40, Aβ42 and Aβ42/40 for the detection of AD in different cohorts. AUC of Aβ40 had lower values than the other biomarkers (Mtp-1 0.686 (0.638 to 0.731); Mtp-2 0.751 (0.711 to 0.788); Paris 0,679 (0.641 to 0.716); SPIN-Barcelona 0.730 (0.667 to 0,787)).

**Additional file 5 : Sup-Figure 3.** Aβ40 in different clinical groups. The Montpellier, Paris and SPIN-Barcelona cohorts displayed a large range of pathological samples from patients with Alzheimer’s disease (AD), cerebral amyloid angiopathy (CAA), frontotemporal degeneration (FTD), mild cognitive impairment (MCI), normal pressure hydrocephalus (NPH), vascular dementia (VASC), other neurological diseases (amytrophic lateral sclerosis, Parkinson’s disease, Lewy Body dementia..) (Other) and control (subjective cognitive impairment). Box plots with median and 25th/75th percentile of CSF Aβ40 values are plotted for the four cohorts (panels A-D). Note that the distribution of the diagnoses differ in the cohorts (see Methods). Non parametric Mann–Whitney U test were performed between the AD and the other clinical groups. *P* values below statistical significance are in red.

## Data Availability

The datasets used for the analyses are available from the corresponding author on reasonable request.

## Abbreviations

AD: Alzheimer’s disease
ADNI: Alzheimer’s Disease Neuroimaging Initiative
APP: Amyloid precursor protein
AUC: Area under the curve
Aβ: Amyloid β
CAA: Cerebral amyloid angiopathy
CI: Confidence interval
CLIA: Chemiluminescence immunoassay
CMRR: Centre Mémoire de Ressources et de Recherche
CSF: Cerebrospinal fluid
FTD: Frontotemporal degeneration
MCI: Mild cognitive impairment
MMSE: Mini-Mental State Examination
MRI: Magnetic resonance brain imaging
NAD: Non-Alzheimer’s disease
NFT: Neurofibrillary tangle
NPH: Normal pressure hydrocephalus
PET: Positron emission tomography
PiB: Pittsburgh compound B
PLM: Paris-Lille-Montpellier
QC: Quality control
ROC: Receiver operating characteristic
SD: Standard deviation
SCI: Subjective cognitive impairment
