# Supplementary material for: Cerebrospinal fluid A beta 1–40 peptides increase in Alzheimer’s disease and are highly correlated with phospho-tau in control individuals
Source: Alzheimers Res Ther. 2020 Oct 2;12:123. doi: 10.1186/s13195-020-00696-1 (PMC7532565; doi:10.1186/s13195-020-00696-1)
Supplement: Supplementary file 1 — Additional file 1 : SupTable 1. Number of patients/samples in each clinical group and Aβ40 detection method used. [file 13195_2020_696_MOESM1_ESM.docx]

## SupTable 1

Number of patients/samples in each clinical group and Aβ40 detection method used.

| **Cohorts** | **n** | **AD/ADNI(+)** | **NAD/ADNI(-)** | **Ab40 Detection method** |
| --- | --- | --- | --- | --- |
| **Montpellier 1 (Mtp-1)** | 400 | 126 | 274 | Fujirebio ELISA IBL |
| **Montpellier 2 (Mtp-2)** | 504 | 220 | 284 | Fujirebio ELISA IBL |
| **Paris** | 624 | 299 | 325 | Fujirebio ELISA |
| **SPIN-Barcelona** | 227 | 79 | 148 | Fujirebio Lumipulse |
| **ADNI-MS** | 400 | 169 | 231 | Mass spectrometry |
| **ADNI-Elecsys** | 311 | 189 | 122 | Roche Elecsys |
