# Supplementary material for: Cerebrospinal fluid A beta 1–40 peptides increase in Alzheimer’s disease and are highly correlated with phospho-tau in control individuals
Source: Alzheimers Res Ther. 2020 Oct 2;12:123. doi: 10.1186/s13195-020-00696-1 (PMC7532565; doi:10.1186/s13195-020-00696-1)
Supplement: Supplementary file 2 — Additional file 2 : Sup-Table 2. Percentage of AD/ADNI(+) patients in the four classes based on the Aβ40 percentile values recorded in the various cohorts; p25: 25th percentile, p25-50: 25th-50th percentile; p50-75: 50th-75th percentile; p75: 75th. [file 13195_2020_696_MOESM2_ESM.docx]

## Sup-Table 2

Percentage of AD/ADNI(+) patients in the four classes based on the Aβ40 percentile values recorded in the various cohorts; p25: 25th percentile, p25-50: 25th-50th percentile; p50-75: 50th-75th percentile; p75: 75th.

| **% AD** | **Aß40 percentile <25th** | **Aß40 percentile 25-50th** | **Aß40 percentile 50-75th** | **Aß40 percentile >75th** |
| --- | --- | --- | --- | --- |
| **Montpellier 1 (Mtp-1)** | 15.8% | 30.4% | 47.4% | 45.6% |
| **Montpellier 1 (Mtp-2)** | 19.0% | 29.0% | 30.0% | 48.0% |
| **Paris** | 21.4% | 36.0% | 56.7% | 60.3% |
| **SPIN-Barcelona** | 38.5% | 38.5% | 54.5% | 60.3% |
| **ADNI-MS** | 34.6% | 62.3% | 73.1% | 73.1% |
| **ADNI-Elecsys** | 29.0% | 44.0% | 43.0% | 53.0% |
