# Supplementary figures and images for: Cerebrospinal fluid A beta 1–40 peptides increase in Alzheimer’s disease and are highly correlated with phospho-tau in control individuals
Source: Alzheimers Res Ther. 2020 Oct 2;12:123. doi: 10.1186/s13195-020-00696-1 (PMC7532565; doi:10.1186/s13195-020-00696-1)

Sup Figure 1

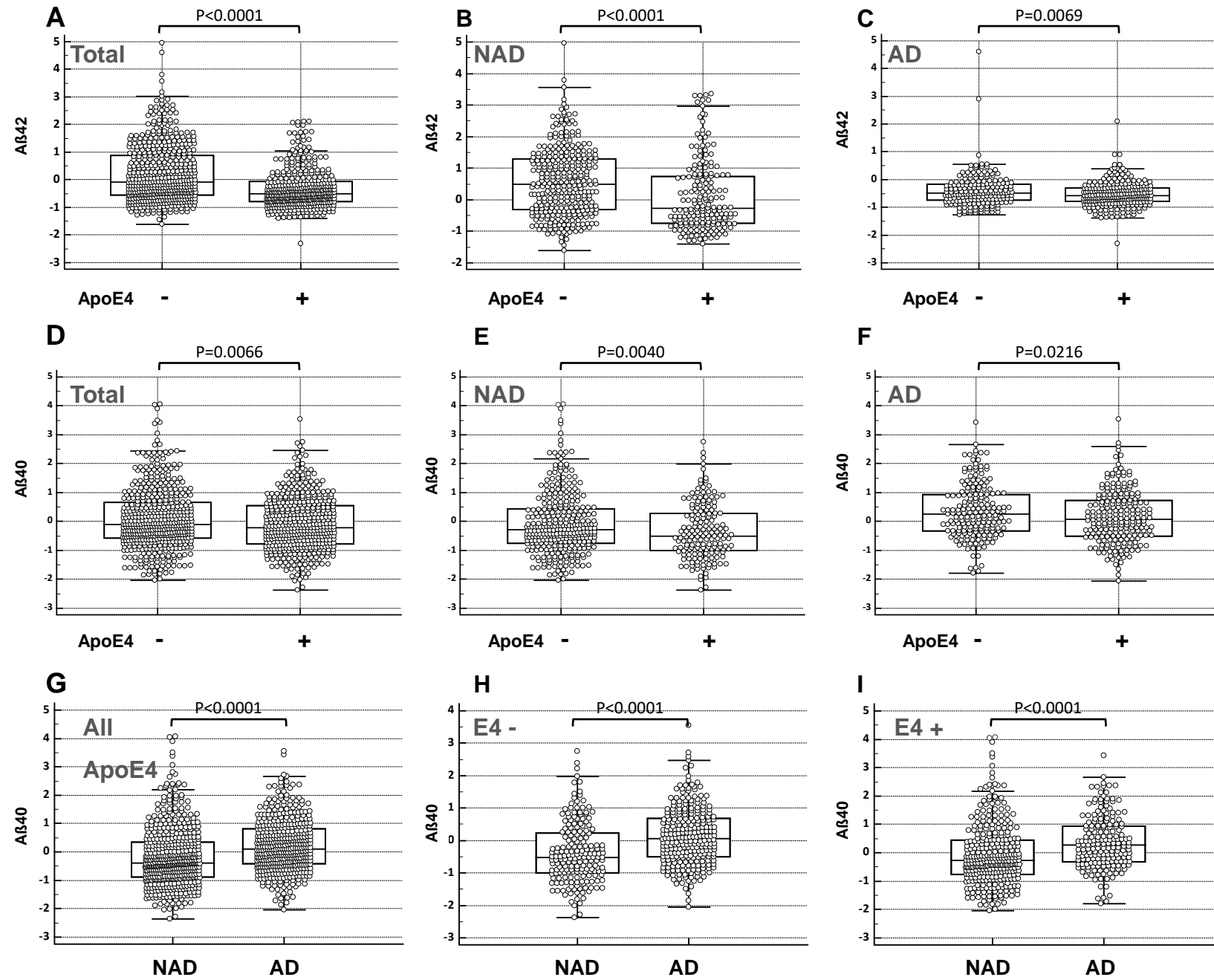

Supplement: Supplementary file 3 — Additional file 3 : Sup-Figure 1. CSF Aβ42 and Aβ40 in Non-AD and AD populations with regards to ApoE status Mean-centered values of CSF Aβ42 (panels A-C) and Aβ40 (panels D-F) in total (all), NAD and AD population with regards to ApoE4 allele presence (+) or absence (-). Panels G-I illustrate the difference in Aβ40 between AD and NAD in the population with the ApoE status determine, in the total (all), ApoE4 allele presence (+) or absence (-). [file 13195_2020_696_MOESM3_ESM.pdf]

Sup Figure 2

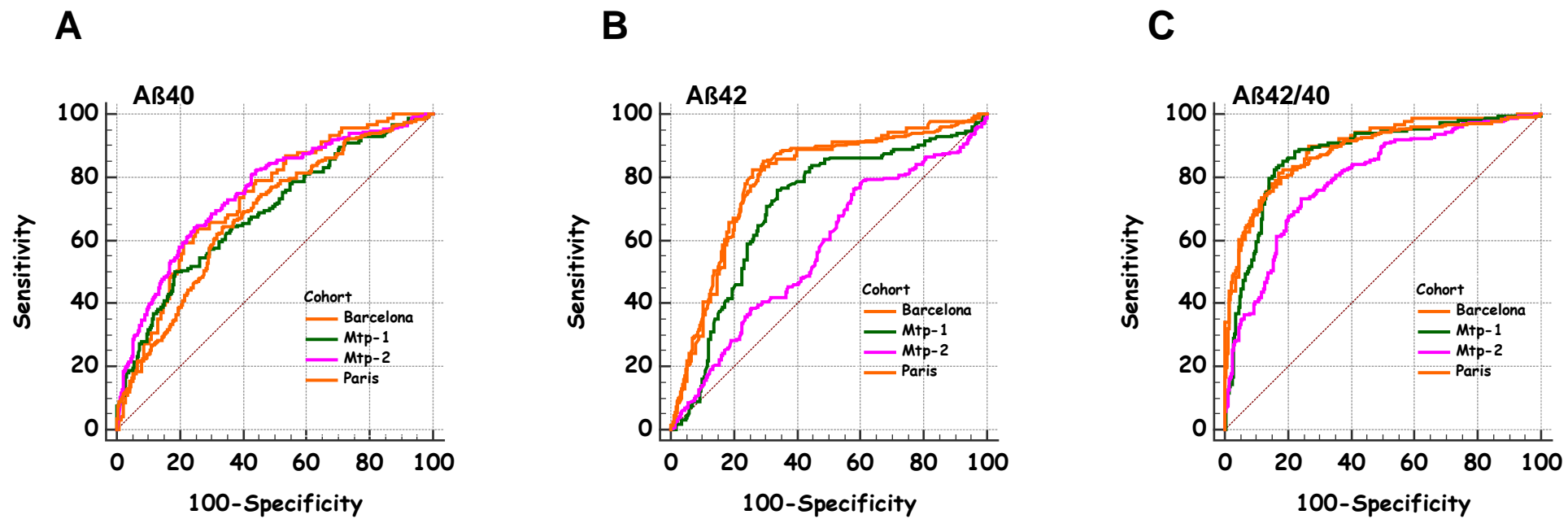

Supplement: Supplementary file 4 — Additional file 4 : Sup-Figure 2. ROC curves of Aβ40, Aβ42 and Aβ42/40 ROC curves of Aβ40, Aβ42 and Aβ42/40 for the detection of AD in different cohorts. AUC of Aβ40 had lower values than the other biomarkers (Mtp-1 0.686 (0.638 to 0.731); Mtp-2 0.751 (0.711 to 0.788); Paris 0,679 (0.641 to 0.716); SPIN-Barcelona 0.730 (0.667 to 0,787)). [file 13195_2020_696_MOESM4_ESM.pdf]

Sup Figure 3

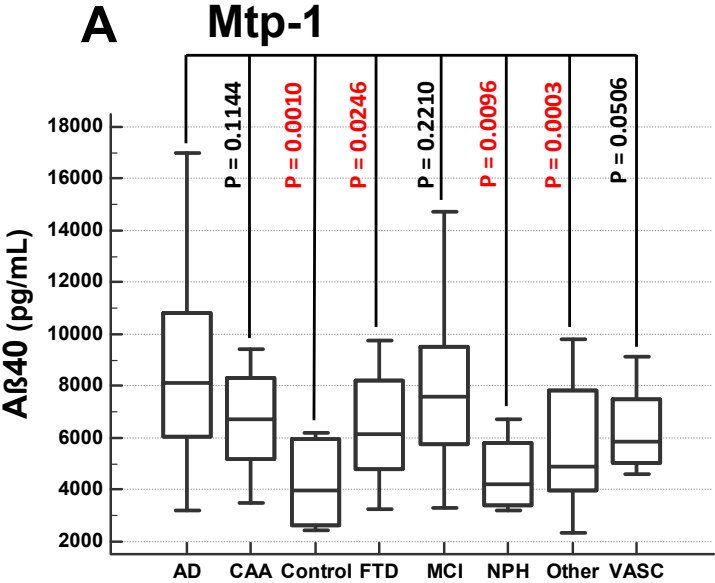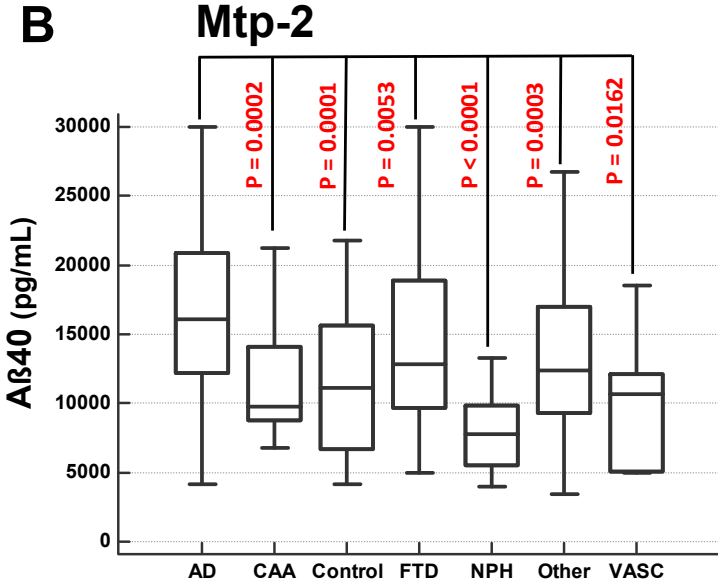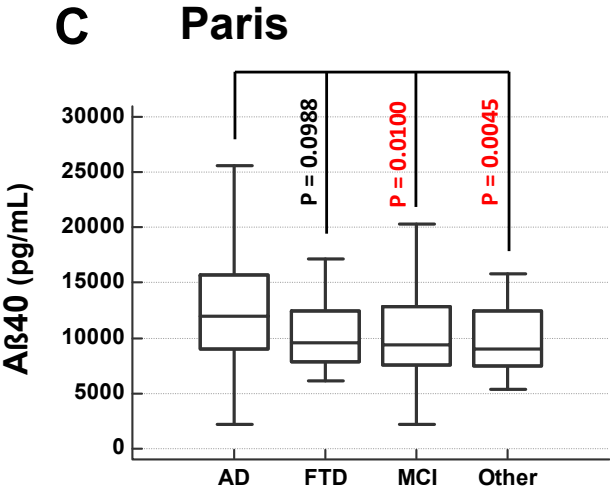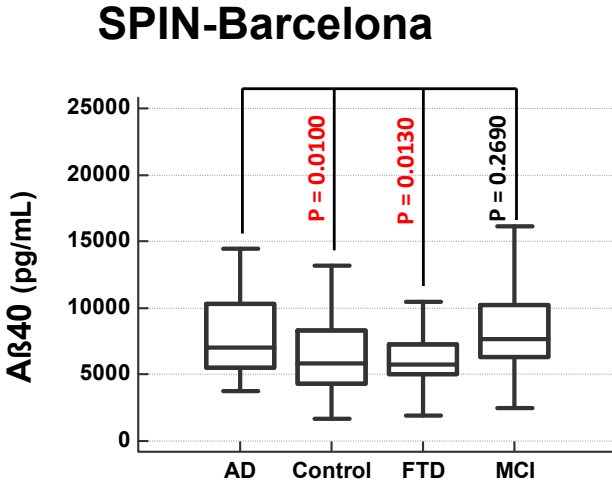

Supplement: Supplementary file 5 — Additional file 5 : Sup-Figure 3. Aβ40 in different clinical groups. The Montpellier, Paris and SPIN-Barcelona cohorts displayed a large range of pathological samples from patients with Alzheimer’s disease (AD), cerebral amyloid angiopathy (CAA), frontotemporal degeneration (FTD), mild cognitive impairment (MCI), normal pressure hydrocephalus (NPH), vascular dementia (VASC), other neurological diseases (amytrophic lateral sclerosis, Parkinson’s disease, Lewy Body dementia..) (Other) and control (subjective cognitive impairment). Box plots with median and 25th/75th percentile of CSF Aβ40 values are plotted for the four cohorts (panels A-D). Note that the distribution of the diagnoses differ in the cohorts (see Methods). Non parametric Mann–Whitney U test were performed between the AD and the other clinical groups. P values below statistical significance are in red. [file 13195_2020_696_MOESM5_ESM.pdf]
